# RISE: A COMMUNITY-BASED ELDER ABUSE AND SELF-NEGLECT RESPONSE INTERVENTION ADDRESSING A CRITICAL SYSTEMS GAP

**DOI:** 10.1093/geroni/igad104.1152

**Published:** 2023-12-21

**Authors:** David Burnes, Karl Pillemer

## Abstract

Knowledge of effective interventions for elder abuse and self-neglect (EASN) is limited. Adult Protective Services (APS) represents the primary agency responsible for receiving reports and investigating suspected cases of EASN in the US. However, APS lacks a distinct, conceptually informed intervention phase to support EASN cases. Based on theory, research, and consultations with stakeholders, RISE was designed to address this intervention gap within overall EASN response systems. Informed by ecological-systems, relational, and client-centered perspectives, RISE is a community-based EASN intervention that integrates core modalities (motivational interviewing, restorative justice, teaming, supported decision making) demonstrating evidence and/or promising results in EASN and other domains. The intervention operates at Relational, Individual, Social, and Environmental levels of ecological influence. Specifically, RISE works with both older adult victims and others, including alleged harmers, their relationships, and strengthens the social supports surrounding them. RISE began as a pilot in two Maine counties, was expanded to the entire state, has been used in over 450 cases, was written into Maine’s 2023 budget, is now being implemented and tested in New Hampshire and Toronto, Canada, and is being expanded to the criminal justice system. This symposium will describe RISE’s development and conceptual underpinnings (presentation 1), findings on implementing “teaming” (social support), an intervention modality (presentation 2), a qualitative evaluation of RISE from the perspective of APS caseworkers (presentation 3), evidence of RISE reducing EASN recidivism (presentation 4), and case studies of implementing RISE (and its restorative justice modality) in the criminal justice diversion context (presentation 5). This is an Abuse, Neglect and Exploitation of Older Persons Interest Group Sponsored Symposium.

